# GAU-PED study for early diagnosis of Gaucher disease in children with splenomegaly and cytopenia

**DOI:** 10.1186/s13023-023-02760-z

**Published:** 2023-06-16

**Authors:** Andrea Pession, Maja Di Rocco, Francesco Venturelli, Barbara Tappino, William Morello, Nicola Santoro, Paola Giordano, Beatrice Filippini, Simona Rinieri, Giovanna Russo, Katia Girardi, Antonio Ruggiero, Eulalia Galea, Roberto Antonucci, Nicola Tovaglieri, Fulvio Porta, Immacolata Tartaglione, Fiorina Giona, Franca Fagioli, Alberto Burlina, Pediatric Gaucher Study Group, Rosamaria Mura, Bambina Russo, Assunta Tornesello, Giuseppe Menna, Delia Russo, Maurizio Caniglia, Sergio Schettini, Daniela Onofrillo, Saverio Ladogana, Adele Civino

**Affiliations:** 1 Pediatric Unit, S. Orsola – Malpighi Clinic, IRCCS Azienda Ospedaliero-Universitaria di Bologna, Via Giuseppe Massarenti 9, 40138 Bologna, Italy; 2 Unit of Rare Diseases, Department of Pediatrics, Giannina Gaslini Institute, Genoa, Italy; 3 Pediatric Nephrology, Dialysis and Transplant Unit, Fondazione IRCCS Ca’ Granda, Ospedale Maggiore Policlinico Di Milano, Milan, Italy; 4 Paediatric Oncology Department, Bari Policlinico General Hospital, Bari, Italy; 5 Interdisciplinary Department of Medicine, Aldo Moro University, Bari, Italy; 6 SSD Oncoematologia Pediatrica U.O. Pediatria, Dipartimento Salute, Donna, Infanzia e Adolescenza Ospedale Infermi Rimini, Rimini, Italy; 7 Pediatric Onco-Hematology Unit, Azienda Ospedaliero-Universitaria Sant’Anna di Ferrara, Ferrara, Italy; 8 Department of Clinical and Experimental Medicine, Paediatric Oncohematology Unit, University of Catania Medical School, 95122 Catania, Italy; 9 Department of Pediatric Hematology and Oncology, Bambino Gesù Children’s Hospital, IRCCS, Piazza Sant’Onofrio, 4, 00165 Rome, Italy; 10 Pediatric Oncology Unit, Fondazione Policlinico Universitario A. Gemelli IRCCS, Università Cattolica Sacro Cuore, 00168 Rome, Italy; 11 Department of Pediatric Onco-Hematology, Pugliese Ciaccio Hospital, Catanzaro, Italy; 12 Pediatric Clinic, Department of Medical, Surgical and Experimental Sciences, University of Sassari, Sassari, Italy; 13 Department of Pediatrics, Niguarda Hospital, Milan, Italy; 14 Children Hospital, Brescia, Italy; 15 Pediatric Hematology Unit, Department of Woman, Child and of General and Specialized Surgery, Università degli Studi della Campania, Naples, Italy; 16 Hematology, Department of Translational and Precision Medicine, Sapienza University of Rome, AOU Policlinico Umberto I, Rome, Italy; 17 Department of Public Health and Paediatrics, Regina Margherita Children’s Hospital, University of Turin, Turin, TO Italy; 18 Division of Inherited Metabolic Diseases, Reference Centre Expanded Newborn Screening, Department of Women’s and Children’s Health, University Hospital, Padua, Italy; 19 Pediatric Oncology Unit, AO Brotzu, Cagliari, Italy; 20 Pediatric Unit, Azienda Ospedaliera Annunziata, 87100 Cosenza, Italy; 21 Pediatric Oncology Unit, Ospedale Vito Fazzi, Piazza Filippo Muratore, 1, 73100 Lecce, Italy; 22 Department of Pediatric Hemato-Oncology, Azienda Ospedaliera di Rilievo Nazionale Santobono Pausilipon, Naples, Italy; 23 Pediatric Hematology and Oncology, ARNAS Civico, Ospedale Di Cristina e Benfratelli, Palermo, Italy; 24 Department of Pediatric and Gynecology, Pediatric Onco-hematology, Perugia Regional Hospital, Perugia, Italy; 25 Department of Obstetrics and Gynaecology, AOR San Carlo, Potenza, Italy; 26 Ospedale Civile, Pescara, Italy; 27 Onco-Hematology Unit, Department of Pediatrics, Casa Sollievo Della Sofferenza, Scientific Institute, San Giovanni Rotondo, Foggia, Italy; 28 Unità di Reumatologia e Immunologia Pediatrica, Ospedale “Vito Fazzi”, Lecce, Italy

**Keywords:** Gaucher disease, Lysosomal storage disease, Splenomegaly, Cytopenia, Thrombocytopenia

## Abstract

**Background:**

Gaucher disease (GD) diagnosis can be delayed due to non-specific symptoms and lack of awareness, leading to unnecessary procedures and irreversible complications. GAU-PED study aims to assess GD prevalence in a high-risk pediatric population and the presence, if any, of novel clinical or biochemical markers associated with GD.

**Materials and methods:**

DBS samples were collected and tested for β-glucocerebrosidase enzyme activity for 154 patients selected through the algorithm proposed by Di Rocco et al. Patients showing β-glucocerebrosidase activity below normal values were recalled to confirm the enzyme deficiency with the gold standard essay on cellular homogenate. Patients tested positive at the gold standard analysis were evaluated through *GBA1* gene sequencing.

**Results:**

14 out of 154 patients were diagnosed with GD, with a prevalence of 9.09% (5.06–14.78%, CI 95%). Hepatomegaly, thrombocytopenia, anemia, growth delay/deceleration, elevated serum ferritin, elevated Lyso-Gb1 and chitotriosidase were significantly associated with GD.

**Conclusions:**

GD prevalence in a pediatric population at high-risk appeared to be higher compared to high-risk adults. Lyso-Gb1 was associated with GD diagnosis. The algorithm proposed by Di Rocco et al. can potentially improve the diagnostic accuracy of pediatric GD, allowing the prompt start of therapy, aiming to reduce irreversible complications.

## Background

According to the most recent classification of inherited metabolic disorders [1], Gaucher disease (GD) is an inborn error of metabolism with an autosomal recessive inheritance that belongs to the sub-category of the Lysosomal Storage Disorders (LSDs). GD is caused by the deficient activity of the β-glucocerebrosidase enzyme (GlcCerase; EC3.2.1.45), which is required for the intra-lysosomal degradation of β-glucosylceramide (GlcCer, glucosylcerebroside), a cell membrane sphingolipid. β-glucocerebrosidase deficiency causes the intracellular accumulation of GlcCer almost exclusively in cells of the mononuclear phagocyte system in the spleen, liver, bone marrow, and lungs. These cells are called 'Gaucher cells' [2]. Recent literature suggests that other downstream metabolic products of glucosylcerebroside, such as β-glucosylsphingosine (GlcSph or lyso-Gb1), may accumulate and play a role in the pathophysiology of the disease [3]. In the vast majority of cases, GD is caused by a homozygous or compound heterozygous mutation in the gene encoding acid beta-glucosidase (*GBA*; 606463) on chromosome 1q22. To date (March 31, 2021), there are 590 known mutations of the *GBA* gene (frame-shift, point, or splice site mutations, deletions, insertions, or recombinant alleles), of which 480 are definitely associated with the onset of Gaucher disease [4].

Regardless of the underlying genetic defect, GD results in a multi-system disorder characterized by phenotypic heterogeneity and a wide clinical spectrum. Three main phenotypes are acknowledged by the contemporary literature. Type 1 (GD1; OMIM # 230800), the non-neuronopathic variant, with prevalent involvement of liver, spleen, bone, and haematological system. Type 2 (GD2; OMIM # 230900), the acute neuronopathic variant which occurs early in childhood, is the most severe form. Type 3 (GD3; OMIM # 231000), the subacute neuronopathic variant, shows clinical onset more typically in childhood or adolescence [5].

All types of GD can be characterized by visceral involvement with splenomegaly and/or hepatomegaly [2]. Other findings might include cytopenia with thrombocytopenia, anemia, leukopenia, bone involvement with Erlenmeyer flask deformity, bone marrow infiltration, bone pain with osteopenia, and systemic symptoms such as growth delay or delayed puberty [6]. The differential diagnosis usually encompasses a wide range of infectious, malignant, and metabolic diseases. These nonspecific symptoms, the phenotypic heterogeneity and the lack of knowledge about the disease often lead to diagnostic delays, and sometimes to a long diagnostic odyssey even children with overt clinical manifestations [7].

LSDs prevalence was reported by different national surveys in the past twenty years [8–11]. Recent data show a variable GD prevalence, ranging from 0.19 per 100.000 in Japan [12] to 1.35 per 100.000 in Australia [13]. Data from newborn screening in northern Italy report a GD incidence of 1 per 12,786 births [14]. The carrier rate is higher in the Ashkenazi Jewish population, 1:16, leading to a GD incidence of 1:850 in this ethnic group [15].

GAU-PED is an observational, non-pharmacological, multicenter, cross-sectional prospective study. The primary objective was to determine the prevalence of GD in a population of paediatric patients (0– ≤ 18 years old) selected by an appropriate diagnostic algorithm who refer to the haematology departments with a clinical history of splenomegaly (with or without hepatomegaly) and thrombocytopenia (and/or anaemia) or with splenomegaly (with or without hepatomegaly) where other causes of splenomegaly were excluded.

Secondary objectives were:

- to assess whether specific risk factors for GD can be identified by comparing clinical and laboratory data of the selected children at risk for GD with those of patients with a confirmed GD diagnosis.
- to validate the diagnostic algorithm proposed by Di Rocco et al. [16].

A preliminary feasibility questionnaire was previously submitted to 41 centers in the context of the AIEOP (Associazione Italiana Ematologia e Oncologia Pediatrica) Study Group, the Italian clinical research consortium in pediatric hematology and oncology with an excellent long-standing track record of clinical trials in children with oncological and hematological diseases. The results of the questionnaire showed that around 700 pediatric patients are evaluated every year, 7% (49 patients) of which are referred for splenomegaly associated with thrombocytopenia. Among them, 61% (30 patients) do not receive a final diagnosis.

## Materials and methods

### Study design

**Fig. 1:**
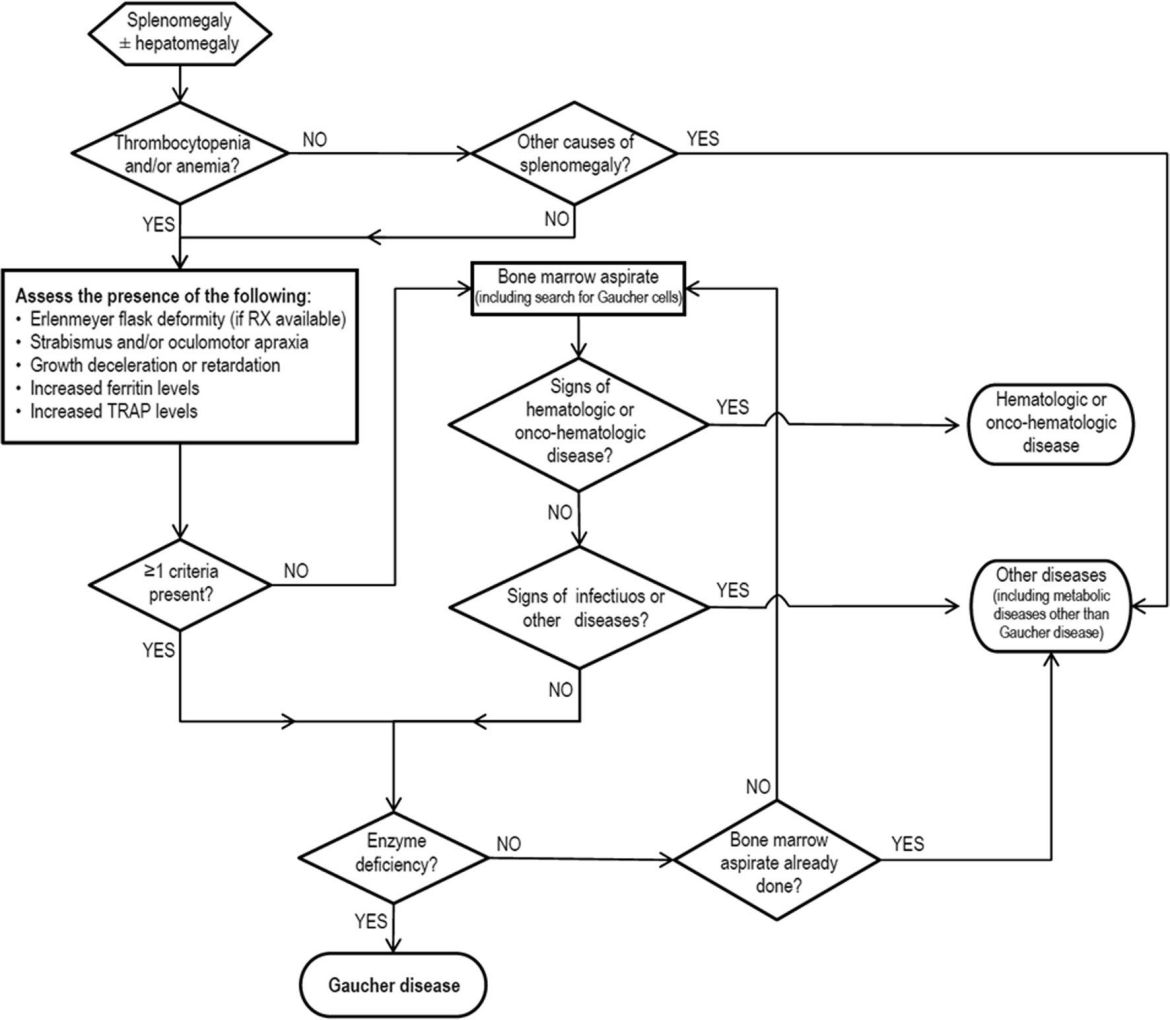
Diagnostic algorithm for childhood splenomegaly and Cytopenia [16]

Enrolment was conducted by 28 AIEOP centers across Italy from July 2015 to July 2020 prior informed consent. 154 patients referred to the pediatric hematology unit of 28 AIEOP centres for splenomegaly with or without hepatomegaly were selected based on the indications contained in the diagnostic algorithm published by Di Rocco et al. [16] (Fig. 1). Inclusion criteria were (1) age ≤ 18 years, (2) splenomegaly with or without hepatomegaly associated with thrombocytopenia and/or anemia, (3) splenomegaly with or without hepatomegaly where other causes of splenomegaly were excluded, (4) informed consent. Exclusion criteria were (1) Age > 18 years, (2) patients already diagnosed with GD, (3) Splenomegaly due to other identified causes: hematologic or onco-hematologic diseases, Infectious diseases, Metabolic diseases other than Gaucher Disease.

For each enrolled patient, a dried blood spot (DBS) sample was collected and tested for the β-glucocerebrosidase enzyme activity. Patients showing enzyme activity on DBS below normal values were recalled to collect a blood sample and confirm the enzyme deficiency using the gold standard essay on leukocytes or EBV-transformed lymphoblasts. Patients with confirmed reduced glucocerebrosidase activity at the gold standard analysis were evaluated through *GBA1* gene sequencing.

Information regarding clinical presentation, age at onset of symptoms, blood tests (particularly cytopenia and GD biomarkers), visceromegaly (detected by clinical examination or by imaging), and bone involvement was collected for every patient.

### Dried blood spot, laboratory assay and molecular analysis

The DBS-based technique was used as the first screening method [17]. Blood collection cards printed with dashed-line circles of 12 mm diameter were provided to all participant centers, along with laboratory instructions for blood collection. All DBS were dried overnight at room temperature and were stored at − 20 °C if not sent within 24 h after the collection and were shipped in a sealed plastic bag to the centralized laboratory to be evaluated. The dried blood spot was processed and analyzed as previously described [17]. A cut-off of 4.4 pmol punch^−1^ h^−1^ was used to assess β-glucocerebrosidase activity on DBS, with a sensitivity of 88.2% and a specificity of 88.5% [18]. Patients showing β-glucocerebrosidase activity lower than 4.4 pmol punch^−1^ h^−1^ on DBS were deemed positive. They were recalled and tested for β-glucocerebrosidase enzyme activity on nucleated cell homogenates (on either leukocytes or lymphoblasts). The normal range used for enzyme activity was 11.6–25.2 nmol/mg/h for leukocytes and 12.6–48.4 nmol/mg/h for lymphoblasts. The normal values for nucleated cells homogenate were obtained by the centralized laboratory using a sample of 50 healthy subjects. If β-glucocerebrosidase activity was either lower than 11.6 nmol/mg/h on leukocytes or 12.6 nmol/mg/h on lymphoblasts, the test was deemed positive. If the enzymatic defect was confirmed, the diagnosis was completed with the molecular analysis of *GBA1* gene. Genomic DNA, collected after written informed consent, was extracted from peripheral blood, lymphoblast cell line or lymphocytes using standard methods. PCR products were purified and directly sequenced using ABI PRISM® 3130 XL Genetic Analyzer (Applied Biosystems, Foster City, CA, USA).

Patients’ data regarding complete blood count (CBC), liver function, serum protein electrophoresis, lipid metabolism (total cholesterol), iron status (serum ferritin, iron, and transferrin), Lyso-Gb1 and chitotriosidase (CHIT) were collected at baseline. All clinical and laboratory data were gathered in a specific case report form (CRF) and collected by the Coordinating Centre at IRCCS Azienda Ospedaliero-Universitaria of Bologna, Italy.

### Statistical analysis

The prevalence of GD and its 95% confidence interval (CI) were calculated based on Clopper-Pearson’s exact method [19]. Demographic, clinical, and laboratory variables of the unaffected patients were compared to those with confirmed GD using the χ^2^ test (or Fisher’s exact test, when appropriate) for categorical variables, whereas the t-test was adopted to compare continuous variables [20]. Z-scores were calculated for β-glucocerebrosidase activity on cellular homogenate to compare the results between patients tested for enzyme activity on leukocytes and lymphoblasts.

Using univariate and multivariate logistic analysis, the role of significant clinical and laboratory variables able to influence the test result were evaluated (Lyso-Gb1, CHIT, ferritin, splenomegaly, hepatomegaly, bone pain, spontaneous fracture, hemorrhage, and strabismus/oculomotor apraxia) [20]. Only variables that were significant in univariate analysis were considered in multivariate (serum ferritin, hepatomegaly, thrombocytopenia, anemia, growth delay).

For some variables (Lyso-Gb1 and CHIT) ROC (Receiver Operating Characteristics) curves were constructed, to measure the sensitivity and the specificity of those variables when determining the diagnostic test result (which is GD) [21, 22].

All p values are two-tailed and values below 0.05 were considered statistically significant.

NCSS 12 (NCSS 2020 Statistical Software. NCSS, LLC. Kaysville, Utah, USA, ncss.com/software/ncss, 2020) and STATA 7.0 (Statacorp, STATA Statistical Software: Release 7.0, Stata Corporation, College Station, TX, 2000) were used for data analysis.

## Results

### Patients’ characteristics

One hundred and fifty-four patients were enrolled in the study (from October 2015 to February 2020).

All patients had splenomegaly at palpation and/or imaging, associated with hepatomegaly in 47 (30.5%) patients. 86 (65.4%) patients had thrombocytopenia (defined as platelet count < 150.000/mmc) and 39 (29.5%) patients showed anemia (defined as haemoglobin < 11 g/dl), with 28 patients (18%) presenting both.

### Laboratory results

**Fig. 2:**
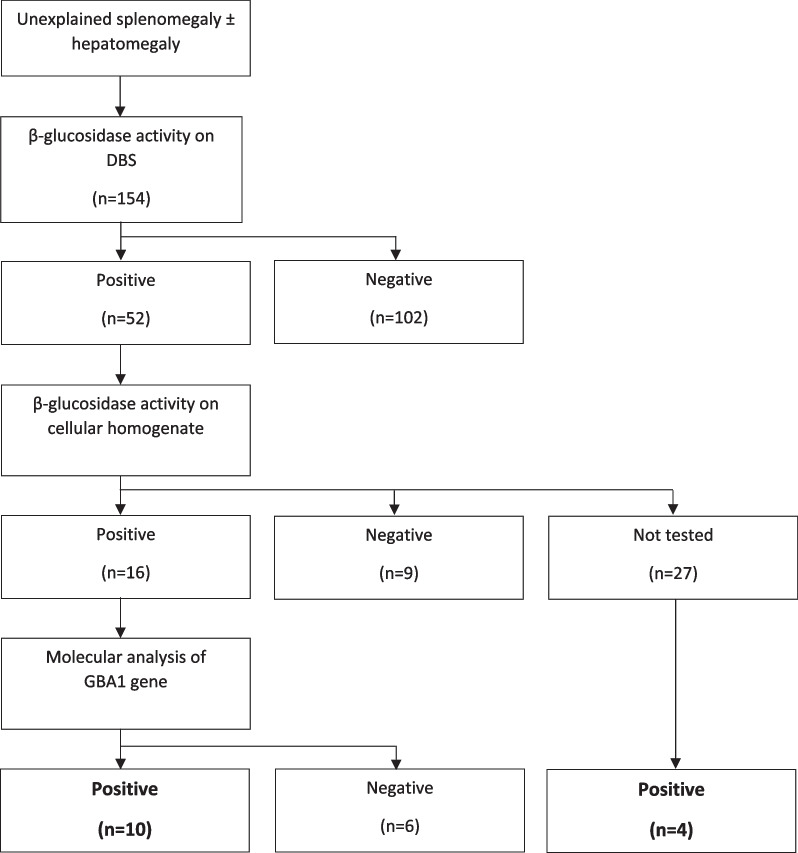
GAU-PED study flowchart. DBS: Dried blood spot

52 patients (33.7%) were found positive at the DBS test, with β-glucocerebrosidase activity values < 4.4 pmol punch^−1^ h^−1^ as previously described. 102 patients (66.3%) had negative DBS test results. Among the DBS-positive patients, 16 (30.8%) showed low β-glucocerebrosidase activity with the enzymatic essay on cellular homogenate (9 patients tested on lymphoblasts and 7 on leukocytes), 9 (5.8%) tested negative and 27 (17.5%) were not tested. The 16 patients tested positive at the enzymatic essay proceeded to *GBA1* gene sequencing, and the diagnosis of GD was confirmed in 10 patients. The remaining 6 patients showed a wild-type *GBA1* gene sequence with normal Lyso-Gb1 values, and the GD diagnosis could not be confirmed. Among the 27 DBS-positive patients that were not tested for β-glucocerebrosidase activity on cellular homogenate, 23 were lost to follow-up and four patients proceeded directly to *GBA1* gene sequencing, ultimately confirming the diagnosis of GD. Thus, 14 out of 154 patients were diagnosed with GD (12 GD1 and 2 GD3), with a prevalence of 9.09% (5.06–14.78%, CI 95%) (Fig. 2). Table 1 reports the characteristics of the 14 GD patients.

**Table 1:** Comprehensive evaluation of 14 patients with molecular confirmation of GD

Pt	DBS value (pmol/punch^−1^/h^−1^)	Conventional enzymatic assay		Lyso-Gb1 (ng/mL)	Genotype	Phenotype
		Leucocytes (nmol/mg/h)	Lymphoblasts (nmol/mg/h)			
1	1.10	1.20	0.40	1921.9	p.Leu483Pro/p.Asp448His	GD3
2	1.19	1.30	NA	NA	p.Leu483Pro/p.Leu483Pro	GD3
3	0.00	1.50	NA	NA	c.115+1G>A/p.Asn227Ser	GD1
4	0.70	1.80	NA	2538.3	p.Asn409Ser/c.115+1G>A	GD1
5	0.90	0.90	NA	1087	p.Asn409Ser/G202R	GD1
6	3.50	7.2	NA	441	p.Asn409Ser/p.Leu483Pro	GD1
7	0.60	NA	1.07	546.8	p.Asn409Ser/p.Asn409Ser	GD1
8	1.19	NA	2.90	NA	p.Ser310Gly/p.Gly234Trp	GD1
9	1.30	4.00	NA	1017.5	p.Asn409Ser/p.Asn409Ser	GD1
10	1.40	1.50	NA	NA	p.P138Lfs*62/p.Asn409Ser	GD1
11	1.30	NA	NA	NA	p.Asn409Ser/p.Asn409Ser	GD1
12	0.90	NA	0.50	328	p.Asn409Ser/p.Arg86*	GD1
13	0.67	NA	NA	NA	p.Asn409Ser/p.Leu483Pro	GD1
14	3.15	NA	NA	1255.9	c.115+1G>A/p.Asn409Ser	GD1

*Pt* patient, *NA* not available, *GD1* Gaucher disease subtype 1, *GD3* Gaucher disease subtype 3

**Fig. 3:**
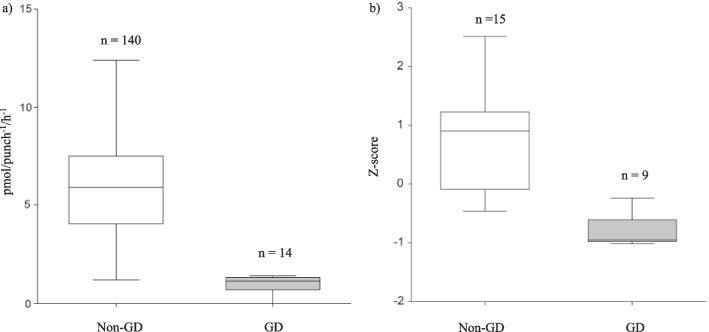
β-glucocerebrosidase enzyme activity comparison between patients with and without GD. **a** β-glucocerebrosidase activity on DBS; **b** β-glucocerebrosidase activity on cellular homogenate (Z-score)

T-test analysis showed a statistically significant difference between β-glucocerebrosidase activity on DBS for patients with and without GD (p < 0.01) (Fig. 3a). Notably, DBS values of patients with confirmed GD differ significantly from those positive at DBS analysis but with wild-type *GBA1* sequence (p < 0.01, data not shown).

Z-score of patients with confirmed GD was found to be significantly lower than Z-score of patients without GD (p < 0.01) (Fig. 3b).

**Fig. 4:**
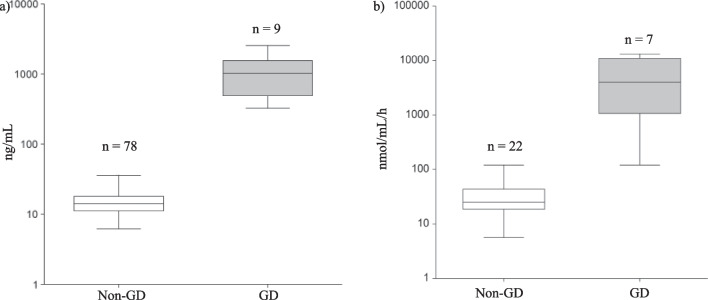
**a** Lyso-GB1 values comparison between patients with or without GD. **b** Chitotriosidase values comparison between patients with or without GD

Clinical (Table 2) and laboratory (Table 3) characteristics of patients with (n = 14) and without (n = 140) confirmed GD diagnosis were compared between patients with confirmed GD (n = 14) and patients with unconfirmed GD (n = 140). Hepatomegaly (p < 0.01), thrombocytopenia (p < 0.05), anemia (p < 0.01), and growth delay or deceleration (p < 0.05) appeared to be significantly associated with GD. Moreover, patients with GD showed significantly higher serum ferritin levels than patients without GD (p = 0.01) and significantly lower hemoglobin values (p < 0.01). Lyso-Gb1 (p < 0.01) (Fig. 4a) and chitotriosidase (CHIT)(p = 0.02) (Fig. 4b) were also significantly increased in GD patients. Multivariate analysis showed that none of the independent variables had any significant impact on the outcome of GD.

**Table 2:** Patients’ clinical features and comparison between patients with or without GD

	Total number of patients (n = 154)	Gaucher patients (n = 14)	Non-Gaucher patients (n = 140)	*P* value
Gender				
Female	47 (30.5%)	6 (42.9%)	41 (29.3%)	
Male	107 (69.5%)	8 (57.1%)	99 (70.7%)	
Age (yrs), mean ± SD	10.1 ± 5.5	10.8 ± 5.3	10 ± 5.5	0.61
Hepatomegaly	47 (30.5%)	11 (78.6%)	36 (25.7%)	** < 0.01**
Thrombocytopenia	86 (55.8%)	12 (85.7%)	74 (52.9%)	** < 0.05**
Anemia	39 (25.3%)	10 (71.4%)	29 (20.7%)	** < 0.01**
Hyperferritinemia	8 (5.2%)	2 (14.3%)	6 (4.3%)	0.26
Bone pain	21 (13.6%)	3 (21.4%)	18 (12.9%)	0.41
Growth delay or deceleration	15 (9.7%)	5 (35.7%)	10 (7.1%)	** < 0.05**
Strabismus and/or oculomotor apraxia	3 (1.9%)	1 (7.1%)	2 (1.4%)	0.25

Statistically significant* p*-values in bold

% denotes the proportion as compared to the respective total number (n) of individuals

**Table 3:** Laboratory tests: comparison between patients with or without Gaucher disease

	Gaucher patients (n = 14)	Non-Gaucher patients (n = 140)	*P* value
β-Glucocerebrosidase activity on DBS (pmol punch^−1^ h^−1^)	1.27 ± 0.94	6.5 ± 4.5	** < 0.01**
β-Glucocerebrosidase activity on cellular homogenate (leukocytes, nmol/mg/h)	2.4 ± 2.2	16.9 ± 4.1	
β-Glucocerebrosidase activity on cellular homogenate (lymphoblasts, nmol/mg/h)	1.2 ± 1.2	9.9 ± 7.2	
β-Glucocerebrosidase activity on cellular homogenate (Z-score)	− 0.49 ± 0.2	0.69 ± 0.86	** < 0.01**
Hb (g/dl)	10.4 ± 1.96	12.3 ± 2.41	** < 0.01**
Plt (number/mmc)	118,357 ± 89,918	167,600 ± 116,771	0.07
WBCs (number/mmc)	6090 ± 3833	9116 ± 29,733	0.27
Serum iron (mcg/dL)	61.6 ± 29	76.4 ± 44	0.14
Serum ferritin (mcg/L)	191.7 ± 134.9	80.9 ± 158.3	**0.01**
Serum transferrin (mg/dL)	231.6 ± 178.1	256.9 ± 100.3	0.68
Serum Total Cholesterol (mg/dL)	117 ± 30.5	129.5 ± 40.2	0.29
ALT (U/L)	40.5 ± 22.4	36.7 ± 73.7	0.68
AST (U/L)	58.6 ± 72.3	42.9 ± 105.4	0.51
Lyso-Gb1 (ng/mL)	1080.1 ± 739.3	14.9 ± 6.5	** < 0.01**
CHIT (nmol//mL/h)	5358.6 ± 4949.8	35.1 ± 30.3	**0.02**

Statistically significant* p*-values in bold

Data are presented as “mean ± standard deviation”

*Hb* haemoglobin, *Plt* platelets, *WBCs* white blood cells, *ALT* alanine transferase, *AST* aspartate transferase, *Lyso-Gb1* β-glucosylsphingosine, *CHIT* chitotriosidase

## Discussion

Differential diagnosis of splenomegaly with or without cytopenia in the pediatric setting is particularly challenging, because of the diverse underlying etiologies and the lack of specific signs or symptoms. A considerable number of these patients are misdiagnosed or remain without a conclusive diagnosis [23]. Although GD is a rare disorder, it is of the utmost importance for the pediatrician to reduce late diagnosis in order to start treatment, addressing current signs and symptoms and preventing serious complications with a high impact on the quality of life (such as bone deformities). Patients are usually referred to the pediatric hematologist for consultation because of the most frequent alterations (thrombocytopenia, anemia, and increased serum ferritin).

Pediatricians sorely need better awareness and better diagnostic tools, even more so if we consider that GD prevalence could be higher than expected. A recent multicentric observational study on an Italian cohort of adult patients reported a prevalence of GD of 3.3% [24] (15 out of 455 patients with splenomegaly and/or thrombocytopenia). Our results indicate that the prevalence of GD in the pediatric population is much higher, with 9.09% (14 out of 154 patients) having confirmed pathological *GBA1* gene mutations. Although 4 out of 14 patients with GD were diagnosed with *GBA1* sequencing directly after DBS positivity, we included them in the patients’ population since our primary objective was to assess the GD prevalence in our cohort. 2 out of 14 patients were diagnosed with GD3. One showed strabismus and supranuclear gaze palsy and the other developed myoclonus.

When we compared laboratory data of GD patients with the rest of the cohort, we found that serum ferritin was significantly elevated in GD patients. This finding is consistent with previous reports from adults [25, 26]. Since this finding is probably related to macrophage activation and the subsequent release of IFs and IL-4 pathway-mediated cytokines [27], it is reasonable to assume that enzyme replacement therapy (ERT) can reduce ferritin levels by modulating inflammatory response [28].

Pediatric patients with confirmed GD also showed significantly lower hemoglobin levels and higher rates of growth delay compared to the rest of the cohort; the hypersplenism and the large mass of Gaucher cells are known factors that exacerbate anemia, whereas osteoclastic and osteoblastic dysfunction is usually the cause of growth delay and, ultimately, bone deformities (mainly femur and tibia) [29].

Notably, Lyso-Gb1 was found to be significantly higher in patients with confirmed GD. Since glucosylsphingosine (Lyso-Gb1) is a deacylated form of glucosylceramide degraded by the glucocerebrosidase enzyme, it accumulates when the enzyme activity is lower. This result is in accordance with recent literature, indicating the Lyso-Gb1 as a sensitive biomarker for the diagnosis of GD in both children and adults [3, 30] and could even play a role in the subtype differentiation [31]. Plasma chitotriosidase (CHIT) levels were also found significantly increased in GD patients; this is consistent with previous findings, where CHIT was consistently elevated in GD patients. CHIT levels might be elevated in the setting of lysosomal storage disorders other than GD, where macrophages participate in the accumulation of storage materials [32–34], but they usually present with lower values. Since Lyso-Gb1 and CHIT are already widely available in clinical laboratories, they could prove useful in the clinical setting for the diagnosis of difficult cases.

Interestingly, six patients with a positive DBS test and positive β-glucocerebrosidase enzymatic test showed a non-pathological *GBA1* gene sequence with wild-type sequences. No heterozygotes were found. One of these patients had only mild CHIT elevation (120 nmol/L), while all patients showed normal Lyso-Gb1 values. Clearly, these six patients represent a dire diagnostic challenge for the pediatric hematologist: to date, they showed no other sign or symptom, and follow-up is ongoing. One patient later showed a decreased value of sphingomyelinase activity on white blood cells, with a potential diagnosis of acid sphingomyelinase deficiency (ASMD), but he remains asymptomatic at follow-up. GD and ASMD (type B and type A/B) may have overlapping clinical presentation: for this reason, they can be tested simultaneously on the same DBS in Italy at present, while this method was not available when the GAU-PED trial started. Lyso-Gb1 was particularly useful in this subset of six patients to resolve the diagnostic doubt and proceed to the follow-up and eventually to other clinical investigations despite the low enzyme activity on DBS and leukocytes. Lyso-Gb1 value in GD diagnosis and follow-up is well known [3], and our findings corroborates its value as a useful biomarker for the screening of GD, even when β-glucocerebrosidase activity is decreased. Paired with the DBS analysis or as second-tier test, LysoGb1 is an important tool to refine GD diagnosis. To further characterize this peculiar patients’ subgroup, saposin C gene sequencing was performed, but all patients carried the wild-type variant. Saposin C was proposed as a potential phenotype modifier for GD [35], but our results were inconclusive.

Indeed, this study has some limitations to consider. Even if great effort went into family counselling to explain the importance of early diagnosis, patients were lost during follow-up or testing, and may be difficult to follow their clinical evolution.

Nevertheless, our observational study clearly showed that (i) GD prevalence is higher among children with unexplained splenomegaly and thrombocytopenia than in similarly affected adults. Children are less likely than adults to have diseases associated with splenomegaly and thrombocytopenia due to acquired causes such as malignancies and chronic liver disease. (ii) Lyso-Gb1 and chitotriosidase dosages could prove useful in the differential diagnosis of pediatric patients with splenomegaly and/or hepatomegaly associated with cytopenia after the most common etiologies were ruled out. When glucocerebrosidase activity assays are equivocal, measurement of plasma lyso-GB1 concentrations or chitotriosidase activity can be confirmatory (Fig. 4). Moreover, the clinical algorithm proposed by Di Rocco et al. was a useful guide for the pediatric hematologist to achieve the diagnosis for a rare and challenging disease such as GD and should be included in diagnostic guidelines issued for pediatric hematologists and general pediatricians.

## Conclusions and future perspectives

This study, for the first time, examines the actual prevalence of GD in a pediatric population at increased risk for GD, such as patients aged 0–18 years with hepatosplenomegaly associated with cytopenia or splenomegaly without other causes and identifies a significant number of patients with GD who have not yet been diagnosed.

The use of the algorithm proposed by Di Rocco et al. can potentially improve the diagnostic accuracy for patients with hematological signs and symptoms, allowing an earlier diagnosis of GD and the prompt beginning of therapy in already symptomatic pediatric patients before the onset of irreversible complications.

The diagnosis of rare diseases poses both clinical and economical challenges: diagnostic algorithms aim to aid the physician to overcome some of these challenges, making good use of the available diagnostic resources. In Italy, a large number of rare metabolic diseases are identified at birth because of the expanded newborn screening programs. Neonatal GD diagnosis on large scale has already been piloted in Northern Italy [36], but it is not yet included in the mandatory newborn screening. Since a population-based screening for GD is not feasible to date, the diagnostic algorithm might represent our best tool to improve GD diagnosis efficiency.

## Data Availability

The dataset used and analysed during the current study is available by the corresponding author on reasonable request.

## Abbreviations

GD: Gaucher disease
DBS: Dried blood spot
LSDs: Lysosomal storage disorders
AIEOP: Associazione Italiana Ematologia e Oncologia Pediatrica
CBC: Complete blood count
CHIT: Chitotriosidase
CRF: Case report form
CI: Confidence intervals
ROC: Receiver operating characteristics
ERT: Enzyme replacement therapy
ASMD: Acid sphingomyelinase deficiency
